# Statins and the Lifetime Outcomes of MASLD: From Evidence Asymmetry to Outcome Convergence

**DOI:** 10.3390/ijms27219407

**Published:** 2026-10-22

**Authors:** Javier Crespo, Marta Alonso-Peña, Carolina Jiménez-González, Lorena Cayón-González, Paula Iruzubieta

**Affiliations:** 1 Cantabria Cohort, Valdecilla Research Institute (IDIVAL), 39011 Santander, Spain; 2 Faculty of Medicine, University of Cantabria, 39011 Santander, Spain; 3 MedicineAI, 28027 Madrid, Spain; 4 Departmento de Anatomía y Biología Celular, Universidad de Cantabria, 39011 Santander, Spain; 5 Clinical and Translational Research in Digestive Diseases, Valdecilla Research Institute (IDIVAL), 39011 Santander, Spainp.iruzubieta@gmail.com (P.I.); 6 Gastroenterology and Hepatology Department, Marqués de Valdecilla University Hospital, 39008 Santander, Spain

**Keywords:** MASLD, MASH, statins, cardiovascular disease, liver-related outcomes, outcome convergence

## Abstract

Metabolic dysfunction-associated steatotic liver disease (MASLD) is a systemic disorder in which cardiovascular, hepatic and oncological outcomes shape long-term prognosis. Statins are particularly relevant because their cardiovascular benefit is firmly established, yet they remain underused in MASLD, while their potential liver-related effects remain uncertain. Observational studies consistently associate statin exposure with slower fibrosis progression, fewer major adverse liver outcomes, lower hepatocellular carcinoma incidence and reduced liver-related mortality. However, direct randomized evidence in MASLD remains limited, and available studies differ substantially in disease stage, exposure, endpoint and follow-up. In this narrative review, we critically examine the cardiovascular, hepatic, oncological, randomized, observational, genetic and pharmacological evidence on statin therapy in MASLD. We aim to clarify the apparent discordance between evidence sources, assess whether statins may modify liver-related outcomes beyond their established cardiovascular benefit, and propose a framework for evaluating multidomain therapeutic effects. We propose outcome convergence as a framework for assessing whether a therapy can improve more than one clinically relevant disease domain while preserving independent causal evaluation of each outcome. Statins should therefore be regarded as a candidate, rather than an established, outcome-convergent therapy.

## 1. Introduction

Metabolic dysfunction-associated steatotic liver disease (MASLD) is the most prevalent chronic liver disease worldwide and is increasingly recognized as a systemic disorder rather than an isolated hepatic condition [1,2]. Its clinical burden extends beyond progression to advanced fibrosis, cirrhosis, hepatocellular carcinoma (HCC), and liver-related mortality, encompassing substantial cardiovascular and metabolic morbidity [2,3,4,5]. Across much of the MASLD spectrum, atherosclerotic cardiovascular disease remains a major determinant of long-term outcomes, while the relative contribution of liver-related events increases with advancing fibrosis and cirrhosis [2,4,6]. Consequently, therapeutic priorities in MASLD cannot be defined solely by liver histology or by the risk of hepatic progression but must account for the competing clinical trajectories that shape lifetime prognosis.

Statins occupy a particularly important position within this multidimensional disease framework. Their capacity to reduce low-density lipoprotein cholesterol and prevent major atherosclerotic cardiovascular events is supported by extensive randomized evidence [7], and current clinical guidelines recommend their use according to cardiovascular risk in patients with MASLD [2,8]. Despite this, statins remain underused in this population [9,10], partly reflecting a persistent legacy of concerns regarding hepatotoxicity and the fragmentation of cardiovascular risk management across specialties. At the same time, an expanding body of observational evidence has associated statin exposure with more favourable liver-related outcomes, including slower fibrosis progression, fewer episodes of hepatic decompensation, lower HCC incidence, and reduced liver-related mortality [11,12,13].

However, the strength of evidence supporting these different effects is highly asymmetric. Cardiovascular benefit is causally established [7], whereas the potential hepatic and oncological effects of statins remain supported predominantly by observational studies, small randomized trials, surrogate endpoints, and studies performed in heterogeneous stages of chronic liver disease [11,12,13,14,15,16]. These evidence streams frequently address different populations, exposures, outcomes, and biological time horizons, making apparent discordance difficult to interpret. Moreover, the distinction between association and causal disease modification is especially important for statins, because treatment duration, adherence, cumulative exposure, healthy-user effects, and survivor selection can all influence observational estimates [17,18,19,20,21,22].

A previous review examined the molecular and hepatic mechanisms potentially underlying the hepatoprotective effects of statins in MASLD [23]. Building on this mechanistic perspective, the present review shifts the focus towards clinically relevant outcomes, critically appraises the causal strength and limitations of different evidence sources, and introduces outcome convergence as a framework for evaluating multidomain therapeutic effects.

This evidence asymmetry makes statins a useful model through which to examine a broader problem in MASLD therapeutics: how should the value of an intervention be judged when it may influence several clinically important outcomes arising from the same systemic disease, but with different levels of causal certainty? In this narrative review, we critically appraise the available evidence on statins in MASLD, integrating randomized, observational, genetic, pharmacological, and trial-registry data to examine their potential effects across cardiovascular, hepatic, oncological, and mortality outcomes. We examine the sources of discordance between these evidence domains, the importance of exposure fidelity and disease stage, and the implications of increasingly layered cardiometabolic and liver-directed treatment strategies. Finally, we propose “outcome convergence” as a framework for evaluating whether a therapy—or a deliberately constructed therapeutic strategy—can meaningfully modify more than one major clinical trajectory, while preserving independent causal evaluation of each outcome.

## 2. Search Strategy and Evidence Appraisal

This narrative review was informed by iterative structured searches of PubMed and ClinicalTrials.gov, supplemented by reference-list tracing and cross-checking of registry records against published reports. Searches were updated through 28 August 2026. The search strategy combined statin-related terms (“statin”, “atorvastatin”, “simvastatin”, “rosuvastatin”, “pravastatin”, “pitavastatin”) with disease-related terms (“MASLD”, “MAFLD”, “NAFLD”, “MASH”, “NASH”, “liver fibrosis”, “cirrhosis”) and outcome-related terms (“cardiovascular events”, “liver-related outcomes”, “hepatic decompensation”, “portal hypertension”, “hepatocellular carcinoma”, “cancer”, “mortality”).

Studies were selected according to their relevance to the principal clinical and causal questions addressed in this review. We considered randomized and non-randomized interventional studies, longitudinal cohorts, target-trial emulations, genetic analyses, and systematic reviews/meta-analyses. Particular attention was given to studies reporting clinically meaningful cardiovascular, hepatic, or oncological outcomes, while trials evaluating histological, imaging, biochemical, or haemodynamic endpoints were included when they informed the potential mechanisms or limitations of statin therapy. Both positive and negative or neutral studies were considered to avoid selectively emphasizing favourable findings. ClinicalTrials.gov records were examined to identify ongoing, completed, terminated, or withdrawn trials relevant to statin therapy in MASLD and chronic liver disease, including studies without published results. Registry information was cross-checked against available publications to assess recruitment, completion status, and outcome reporting. Evidence was appraised according to causal certainty, MASLD-specific relevance, statin-exposure fidelity, time horizon, independence of the data-generating population, and compatibility between the study estimand and the clinical question.

## 3. The Dual Statin Paradox

MASLD presents a dual statin paradox: statins remain underused where their evidence is firmly established, yet their potential value is most actively debated where evidence remains uncertain. The first paradox is one of implementation. Cardiovascular disease remains a major determinant of morbidity and mortality across much of the MASLD spectrum, and randomized evidence has conclusively shown that statin-mediated LDL-cholesterol lowering reduces major vascular events [7]. Accordingly, contemporary European and AASLD guidance recommends statin therapy according to cardiovascular risk and considers stable MASLD, including compensated cirrhosis, compatible with statin use [2,8]. Yet treatment remains suboptimal. In a population-based analysis applying contemporary cardiovascular-risk criteria, 33% of individuals with MASLD who had an indication for statin therapy were untreated, compared with 19% of those without MASLD [9]. This treatment gap is clinically relevant irrespective of whether statins exert any hepatic effect. Historical reluctance to prescribe statins in chronic liver disease was driven largely by concerns about hepatotoxicity, but this explanation no longer fully accounts for current practice. In US population data, MASLD became progressively less predictive of statin non-use over time [10], while a primary-care cohort found that chronic liver disease itself was not independently associated with lower prescribing, although alanine aminotransferase concentrations above 45 U/L remained associated with therapeutic inertia [24]. Hepatic steatosis also does not appear to materially attenuate LDL-cholesterol lowering after statin initiation [25]. The current challenge is therefore less one of safety than of implementation, likely compounded by fragmented responsibility for cardiovascular risk across hepatology, diabetology, primary care and cardiovascular medicine.

The second paradox runs in the opposite direction. Beyond their established cardiovascular indication, statins have repeatedly been associated with a more favourable hepatic trajectory. Longitudinal studies have linked statin exposure to slower progression of liver stiffness and fewer liver-related events [11]; large MASLD cohorts have reported lower risks of decompensation, HCC and liver-related mortality [12]; and greater cumulative exposure has been associated with a lower risk of primary liver cancer [13]. These observations are consistent and biologically plausible, but consistency does not establish causality. Direct randomized evidence in MASLD remains limited and is largely confined to biochemical, imaging or histological endpoints, whereas trials evaluating hard clinical outcomes have predominantly enrolled patients with mixed-aetiology cirrhosis. Current guidance therefore appropriately distinguishes the established cardiovascular indication from the still-unproven possibility of liver-related benefit [2].

The paradox is therefore epistemological as well as therapeutic: causal certainty is greatest where implementation remains incomplete, whereas the most ambitious hepatic and oncological hypotheses rest on substantially weaker evidence. The same drug, within the same systemic disease, can therefore be supported by near-maximal causal certainty for one major outcome and substantial uncertainty for another. Persistent observational signals should neither be promoted to causal proof nor dismissed simply because the definitive experiment has not yet been performed.

Statins are not unique in having the potential for multidomain benefit. Semaglutide, for example, has demonstrated randomized cardiovascular-event reduction in people with overweight or obesity and established cardiovascular disease [26], as well as randomized histological efficacy in MASH with F2–F3 fibrosis [27]. What makes statins particularly informative is not therapeutic primacy, but the asymmetry of their evidence base: decades of proven cardiovascular benefit, extensive population exposure, low cost, repeated hepatic and oncological signals, persistent undertreatment despite an established indication, and the absence of a definitive causal evaluation spanning the major outcomes of MASLD.

## 4. One Disease, Multiple Clinical Destinies: From Pleiotropy to Outcome Convergence

MASLD is a systemic disease in which cardiovascular events, major adverse liver outcomes (MALO), HCC, extrahepatic malignancies, and death compete over the patient’s lifetime. Their relative importance varies with age, metabolic phenotype, fibrosis stage, baseline risk, and time horizon. Contemporary heart-liver frameworks appropriately recognize that metabolic, cardiovascular and liver-directed therapies should no longer be considered within isolated specialty silos [3,28]. However, relative risk does not directly translate into absolute therapeutic opportunity. In steatotic liver disease, cardiovascular events may remain more frequent than MALO over clinically relevant periods [4], and substantial cardiovascular risk persists even in patients with significant fibrosis [6]. MASLD is also associated with an increased risk of extrahepatic malignancy [5]. Thus, the hierarchy of clinically relevant outcomes evolves continuously rather than shifting abruptly from cardiovascular to liver-related disease.

Heart-liver co-management and outcome convergence address complementary but distinct questions. Co-management concerns how the hepatic, metabolic and cardiovascular dimensions of MASLD should be treated together. Outcome convergence concerns how therapeutic evidence should be interpreted when an intervention may influence more than one major clinical trajectory. In this sense, co-management integrates care, whereas outcome convergence integrates evidence across clinically relevant outcomes [3,28].

Outcome convergence should also be distinguished from pleiotropy. Statins affect cholesterol metabolism as well as pathways potentially involved in endothelial function, inflammation, fibrogenesis and carcinogenesis, but multiple biological effects do not in themselves establish multidomain clinical benefit. Pleiotropy describes biological action; outcome convergence requires demonstration of clinically meaningful effects across distinct outcome domains. Changes in LDL cholesterol, aminotransferases, liver fat or inflammatory biomarkers therefore cannot be considered evidence of outcome convergence on their own.

We define outcome convergence as an evidence-based framework for evaluating the capacity of a therapeutic intervention—or of a deliberately constructed treatment strategy—to improve two or more major clinical outcomes arising from the same systemic disease, with the evidentiary strength for each outcome and, where relevant, the causal contribution of each therapeutic component assessed independently. Unlike integrated cardiometabolic management, which focuses on coordinating care, or multidomain risk reduction, which describes a therapeutic objective, outcome convergence focuses on the demonstration and interpretation of clinical effects across distinct disease trajectories. It also differs from pleiotropy, which refers to multiple biological actions without necessarily implying clinical benefit. Its evaluation requires predefined domain-specific outcomes, appropriate causal assessment, and consideration of absolute benefit, disease stage and competing risks. Within this framework, a candidate outcome-convergent therapy has established benefit in at least one major clinical domain together with a credible signal in another; an established outcome-convergent therapy requires causal evidence of clinically meaningful benefit across at least two domains.

Importantly, the framework must capture divergence as well as convergence. Statins illustrate this directly: their cardiovascular benefit is unequivocal [7], yet large-scale randomized evidence also shows a small increase in incident diabetes and worsening glycaemia, predominantly among individuals already predisposed to dysglycaemia [29]. Multidomain evaluation must therefore preserve benefit, neutrality and cross-domain harm rather than reducing them to a single claim of systemic benefit.

The clinical value of outcome convergence is necessarily patient- and stage-dependent. In early MASLD, cardiovascular prevention may account for the greatest absolute benefit; with advancing fibrosis, MALO and HCC become increasingly relevant, although cardiovascular risk often remains substantial [4,6]. The appropriate unit of evaluation is therefore not the organ or the specialty in which a drug was developed, but the set of major outcomes that treatment may modify in a given patient over time.

Age further modifies the relationship between cardiovascular and hepatic risk in MASLD. Advancing age is associated with cumulative metabolic and vascular injury and may influence both fibrosis progression and subclinical atherosclerosis. Carotid intima-media thickness (cIMT), a marker of subclinical vascular changes, may provide complementary information on this age-related vascular burden. In a small observational study of patients with obesity-related MASLD, age and circulating interleukin-15 were independently associated with cIMT [30]. Although statins have been shown to attenuate cIMT progression in randomized cardiovascular studies [31], whether these vascular effects parallel or predict hepatic benefits in MASLD remains unknown. Age-related differences in baseline risk, treatment exposure and survival may also influence observational estimates of statin-associated liver outcomes, reinforcing the need for age-stratified analyses and independent causal evaluation across outcome domains.

**Figure 1:**
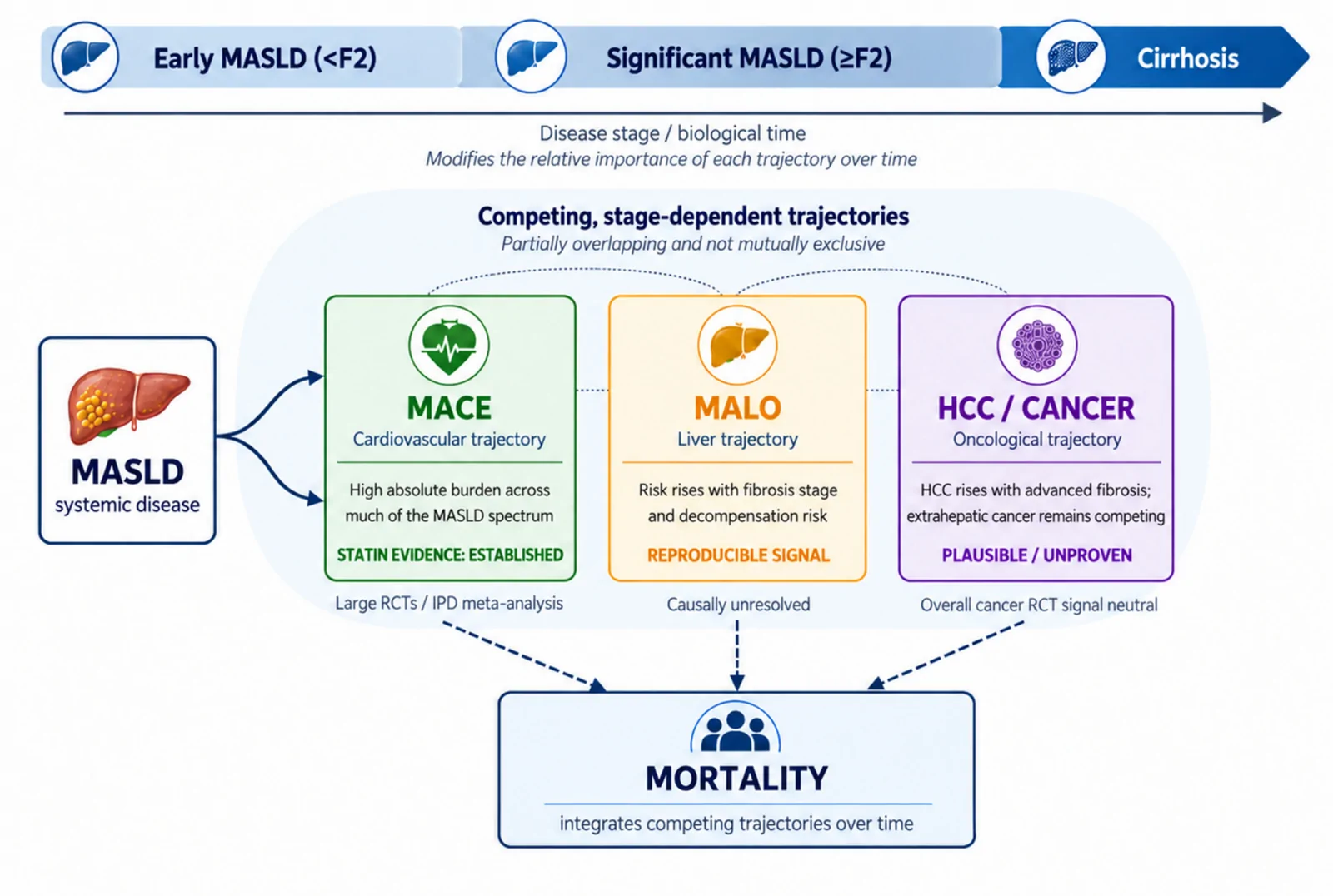
**MASLD as competing lifetime trajectories: evidence asymmetry across domains. **MASLD gives rise to cardiovascular, hepatic, oncological and mortality outcomes whose relative importance changes with disease stage, from early MASLD (<F2) to significant MASLD (≥F2) and cirrhosis. Cardiovascular events remain a major source of morbidity across much of the disease spectrum, whereas the risk of major adverse liver outcomes (MALO) and hepatocellular carcinoma (HCC) increases with advancing fibrosis. Statin-mediated reduction in major adverse cardiovascular events (MACE) is supported by robust randomized evidence, while associations with slower liver disease progression, fewer MALO, lower HCC incidence and liver-related mortality remain observationally consistent but causally unproven. Mortality integrates these competing trajectories over time, underscoring the need to evaluate therapeutic benefit according to disease stage, baseline risk and time horizon. Abbreviations: HCC, hepatocellular carcinoma; IPD, individual participant data; MACE, major adverse cardiovascular events; MALO, major adverse liver outcomes; MASLD, metabolic dysfunction-associated steatotic liver disease; RCT, randomized controlled trial.

Statins provide an unusually mature test case. Their cardiovascular benefit is established [2,7], whereas longitudinal MASLD studies consistently associate statin exposure with slower fibrosis-related progression, fewer liver-related events and lower liver-related mortality [11,12], while repeated observational analyses suggest a lower risk of HCC [12,13]. These signals are clinically coherent but causally unequal. Cancer further illustrates the need for domain-specific adjudication: randomized statin trials involving approximately 175,000 participants did not reduce overall cancer incidence [32], limiting broad anticancer claims without resolving the more specific hypothesis of HCC prevention in high-risk MASLD. Statins should therefore be regarded as a candidate, rather than a demonstrated, outcome-convergent therapy. Figure 1 summarizes this evidence asymmetry across competing lifetime outcomes.

## 5. What Does the Evidence Actually Show? A Gradient of Certainty

The statin literature in MASLD is often described as conflicting, but the apparent discordance largely reflects the fact that different studies address different questions. Populations, disease stage, exposure definitions, comparators, follow-up, endpoints and causal estimands vary substantially across the evidence base. Rather than a binary positive-versus-negative literature, the field is better understood as a gradient of causal certainty and MASLD-specific relevance. Table 1 summarizes the principal evidence architectures and the narrowest inference each can support, while Table 2 provides a quantitative comparison of selected randomized trials and observational cohorts, including study populations, statin exposure, follow-up, and principal effect estimates.

The highest-certainty evidence concerns cardiovascular outcomes. Randomized trials and individual-participant meta-analyses have established that statins reduce major vascular events [7], and current MASLD guidance supports their use according to cardiovascular indication [2]. This evidence stands independently of any potential hepatic effect.

By contrast, the liver-related evidence is dominated by observational data. Across histological, population-based, primary-care, biobank, and longitudinal VCTE cohorts, statin exposure has been associated with more favourable fibrotic phenotypes, slower progression to high-risk FIB-4, less liver-stiffness progression, and fewer liver-related events [11,33,34,35]. Large cohorts have also linked statin use to lower risks of decompensation, cirrhosis, HCC, and liver-related mortality [12,36,37,38].

The HCC signal is particularly persistent. In 272,431 adults with MASLD, statin use was associated with a substantially lower risk of HCC after multivariable adjustment [14]. Similar associations have been reported in large Veterans Affairs and Korean cohorts, with greater cumulative exposure linked to lower primary liver-cancer risk and fewer liver-related events [13,39,40]. Mortality analyses provide additional support [12,33,38,41], and quantitative syntheses generally reproduce the favourable direction of association for HCC and chronic liver outcomes [42,43], although an umbrella analysis reported a statistically neutral estimate in MASLD with type 2 diabetes and substantial heterogeneity [44].

**Table 1:** **Strength and relevance of the evidence on statins in MASLD.** Summary of the main evidence streams evaluating statins in MASLD, highlighting the clinical question addressed, the strength and directness of the evidence, and the valid inference supported by each study design. Cardiovascular benefit is established, whereas liver disease modification and HCC prevention remain causally unresolved.

Evidence Type	Representative Evidence	Main Question/Outcome	Strength and Relevance of Evidence	Valid Inference
Cardiovascular RCTs/meta-analysis	CTT [7]	MACE	Very high causality; indirect for liver modification	Statins causally reduce cardiovascular events irrespective of liver-specific effects.
Direct MASLD RCTs and related metabolic-risk trials	Nelson [15]; Braun [45] (non-MASLD-selected); STAT-NASH [46]	Liver fat/histological MASH-fibrosis	Direct MASLD evidence limited; Braun et al. provide indirect evidence	Direct randomized evidence is limited; hepatic disease modification in MASLD remains unresolved.
MASLD RCTs without isolated statin effect	Athyros [47]; Foster [48]; CONTROL [49]; ESSENTIAL [50]	Liver/metabolic endpoints	Direct MASLD evidence, limited statin attribution	Benefits of treatment strategies cannot be attributed specifically to statins.
Longitudinal observational MASLD and broader population-based cohort	Zhou [11]; Schreiner [35]; Vell [36] (broader population cohorts); Yun [12]	Fibrosis progression, MALO, mortality	High clinical relevance; limited causal certainty	Consistent favourable liver associations, but no proof of MASLD-specific disease modification.
Observational HCC cohorts	Karachaliou [13]; Zou [14]; Kim [39]; Yun [12]	Incident HCC	Reproducible observational evidence	Lower HCC incidence is consistently associated with statin exposure; chemoprevention remains unproven.
Randomized cirrhosis trials	Abraldes [16]; BLEPS [51]; StatLiver [52]; LIVERHOPE [53]; Glal [54]; Rana [55]	Portal pressure, decompensation, mortality	Randomized but low MASLD directness	Biological and clinical signals are heterogeneous and cannot establish MASLD-specific disease modification.
Decompensated cirrhosis safety RCT	LIVERHOPE-SAFETY [56]	Safety	High	The therapeutic window narrows with advanced hepatic dysfunction and higher doses.
Target-trial emulation	Tapper [57]; Choi [58]	Clinical outcomes under emulated treatment strategies	Moderate causal alignment	Reduces avoidable observational biases but cannot eliminate unmeasured confounding.
Drug-target Mendelian randomization	Li [59]; Liang [60]; Ding [61]; Ma [62]	MASLD/HCC genetic proxies	Indirect pharmacological relevance	Genetic evidence constrains but neither proves nor excludes pharmacological benefit.

Abbreviations: CTT, Cholesterol Treatment Trialists’ Collaboration; HCC, hepatocellular carcinoma; MACE, major adverse cardiovascular events; MALO, major adverse liver outcomes; MASLD, metabolic dysfunction-associated steatotic liver disease; MASH, metabolic dysfunction-associated steatohepatitis; NASH, non-alcoholic steatohepatitis; RCT, randomized controlled trial.

**Table 2:** **Key randomized trials and observational cohorts evaluating statins and liver-related outcomes.**

Study/Design	Population/Disease Stage/*N*	Statin Treatment or Exposure	Follow-Up	Main Results
**RANDOMIZED TRIALS**				
Nelson et al. [15]	Biopsy-confirmed MASLD; *n* = 16	Simvastatin vs. placebo	12 months	Histology/aminotransferases: no significant improvement in steatosis, inflammation or fibrosis (10 repeat biopsies).
Braun et al. [45]	Insulin-resistant men with abdominal obesity; *n* = 50 (not selected for MASLD)	Pitavastatin 4 mg/day vs. placebo	6 months	Primary: liver fat difference in change −1 percentage point (95% CI −5 to 3; *p* = 0.56). No improvement in clamp-measured insulin sensitivity.
BLEPS, Abraldes et al. [51]	Cirrhosis after variceal bleeding, mixed aetiologies; *n* = 158 randomized (147 analyzed)	Simvastatin 20→40 mg/day + standard care vs. placebo + standard care	Up to 24 months	Primary (rebleeding or death): HR 0.82 (95% CI 0.47–1.43), NS. Secondary mortality: HR 0.39 (0.15–0.99).
StatLiver, Kronborg et al. [52]	Cirrhosis with portal hypertension, mixed aetiologies; *n* = 78	Atorvastatin 10–20 mg/day vs. placebo	6 months treatment	Hospital admissions: no difference (*p* = 0.97); liver-related admissions: no difference (*p* = 0.61).
LIVERHOPE, Pose et al. [53]	Decompensated cirrhosis, mixed aetiologies; *n* = 237 analyzed	Simvastatin 20 mg/day + rifaximin vs. placebo	12 months	Primary (ACLF): SHR 1.23 (95% CI 0.65–2.34). Secondary death: SHR 1.12 (0.56–2.26).
**OBSERVATIONAL COHORTS**				
Zhou et al. [11]	MASLD across stiffness stages; *n* = 7988 (17% cACLD)	Statin users vs. non-users (40.5% users at baseline)	Median 4.6 years	Liver-related events: aHR 0.38 (95% CI 0.27–0.54); stiffness progression without cACLD: aHR 0.45 (0.34–0.59).
Yun et al. [12]	MASLD/MetALD; *n* = 516,575	Statin users vs. non-users	Median 10.1 years	Primary LREs: aHR 0.64 (95% CI 0.61–0.68); HCC: aHR 0.52 (0.47–0.58).
Zou et al. [14]	MASLD; *n* = 272,431	Statin initiation vs. non-use; IPTW	Median 1.92 years	Incident HCC: IPTW HR 0.47 (95% CI 0.36–0.60); FIB-4 subcohort HR 0.44 (0.30–0.65).
Vell et al. [36]	UKB/PMBB/TriNetX, no prior liver disease; total *n* = 1,785,491; UKB *n* = 205,057	Regular statin use vs. non-use	UKB mean 11.9 years	UKB incident liver disease: HR 0.85 (95% CI 0.78–0.92); HCC: HR 0.58 (0.35–0.96); liver-related death: HR 0.72 (0.59–0.88).

Abbreviations: ACLF, acute-on-chronic liver failure; aHR, adjusted hazard ratio; cACLD, compensated advanced chronic liver disease; CI, confidence interval; FIB-4, Fibrosis-4 index; HCC, hepatocellular carcinoma; HR, hazard ratio; IPTW, inverse probability of treatment weighting; LREs, liver-related events; MASLD, metabolic dysfunction-associated steatotic liver disease; MetALD, MASLD with increased alcohol intake; SHR, subdistribution hazard ratio; UKB, UK Biobank; PMBB, Penn Medicine Biobank.

Taken together, these studies describe a coherent observational continuum from slower fibrosis-related progression to fewer MALO, lower HCC incidence and reduced liver-related mortality [9,11,12,13,14,33,34,35,36,37,38,39,40,41,42,43,44]. However, coherence does not establish causality. The apparent volume of supporting evidence also exceeds the volume of truly independent evidence, because multiple publications arise from the same data environments, including Korean insurance datasets, UK Biobank, Veterans Affairs cohorts and Rotterdam Study. Replication should therefore be judged at the level of independent data-generating populations rather than publication count (Supplementary Table S1).

Randomized evidence directly addressing MASLD remains limited. Most trials have been small, short, surrogate-based, or unable to isolate the statin effect. Athyros et al. evaluated atorvastatin within a multifactorial strategy [47], while the St Francis trial combined atorvastatin with antioxidants [48]. A small placebo-controlled simvastatin trial did not demonstrate histological improvement [15], and pitavastatin did not significantly reduce liver fat or improve insulin sensitivity over six months [45]. CONTROL showed that atorvastatin can mitigate obeticholic acid-associated dyslipidaemia but did not test hepatic disease modification [49]. In ESSENTIAL, both groups received rosuvastatin, precluding isolation of a statin-specific effect [50], while a comparative trial of rosuvastatin, N-acetylcysteine and vitamin E remained based on surrogate endpoints [63]. STAT-NASH is more directly aligned with the disease-modification question, randomizing statin-naïve participants with biopsy-confirmed MASH and fibrosis to atorvastatin 40 mg or placebo for 96 weeks, but its planned sample of approximately 70 participants limits its ability to address hard clinical outcomes [46].

Randomized cirrhosis trials are more clinically consequential but less directly relevant to precirrhotic MASLD. Early studies demonstrated portal-haemodynamic effects with simvastatin [16,64], whereas subsequent trials produced mixed results. BLEPS did not reduce rebleeding, although a secondary survival signal emerged [51], and other proof-of-concept studies reported variable haemodynamic responses [65,66]. LIVERHOPE-SAFETY showed that toxicity in decompensated cirrhosis is strongly influenced by dose and hepatic reserve [56]. StatLiver found no clear clinical benefit of atorvastatin [52], and LIVERHOPE-EFFICACY was neutral for its primary outcome with simvastatin plus rifaximin [53]. Conversely, two recent single-centre trials reported fewer recurrent decompensations with atorvastatin [54] and improved long-term survival after variceal bleeding with simvastatin in open-label design [55]. A 2026 meta-analysis of randomized cirrhosis trials suggested possible portal-pressure and clinical benefit, but trial-sequential analysis indicated that evidence remains underpowered [67].

These trials should not be interpreted as if they addressed a single causal question. Short-term portal-pressure reduction, prevention of variceal rebleeding, recurrent decompensation, mortality in advanced cirrhosis, and histological modification of F2–F3 MASH represent distinct estimands. Post hoc analysis of PROSPER provides additional randomized but indirect evidence in older adults at risk of liver fibrosis [68]. HCC-interception research remains even less mature: a phase II simvastatin trial reduced IL-6 but not the prespecified AFP-L3% endpoint [69]. Biological activity is not equivalent to chemoprevention, and no completed randomized trial has shown prevention of incident HCC specifically in MASLD.

Genetic evidence addresses yet another causal question. Drug-target Mendelian randomization has not provided convincing evidence that genetically proxied HMGCR inhibition reduces susceptibility to MASLD or liver fat [59]. This finding is relevant but not directly equivalent to pharmacological treatment initiated after MASH and fibrosis are already established. Genetic analyses of liver cancer are also inconsistent, with broad liver-cancer and more recent drug-target studies yielding no single stable conclusion and incomplete colocalization support [60,61,62]. Because MASLD susceptibility, fibrosis progression, MALO prevention and HCC prevention are distinct estimands [59,60,61,62,70], genetic evidence constrains the hypothesis more than it resolves it.

The key unanswered questions therefore remain clinically important: whether statins directly slow fibrosis in established MASH, prevent MALO specifically in MASLD, prevent incident HCC, lower extrahepatic cancer risk, or provide multidomain benefit beyond their established cardiovascular effects. The optimal molecule, intensity, cumulative exposure and responder phenotype are also unknown. Safety is no longer the principal uncertainty in non-cirrhotic MASLD or compensated disease [2], although the therapeutic window narrows in decompensated cirrhosis [56], and the modest increase in diabetes risk remains relevant within a multidomain framework [29]. Table 3 summarizes the practical implications across the MASLD spectrum.

## 6. The Sources of Discordance

**Figure 2:**
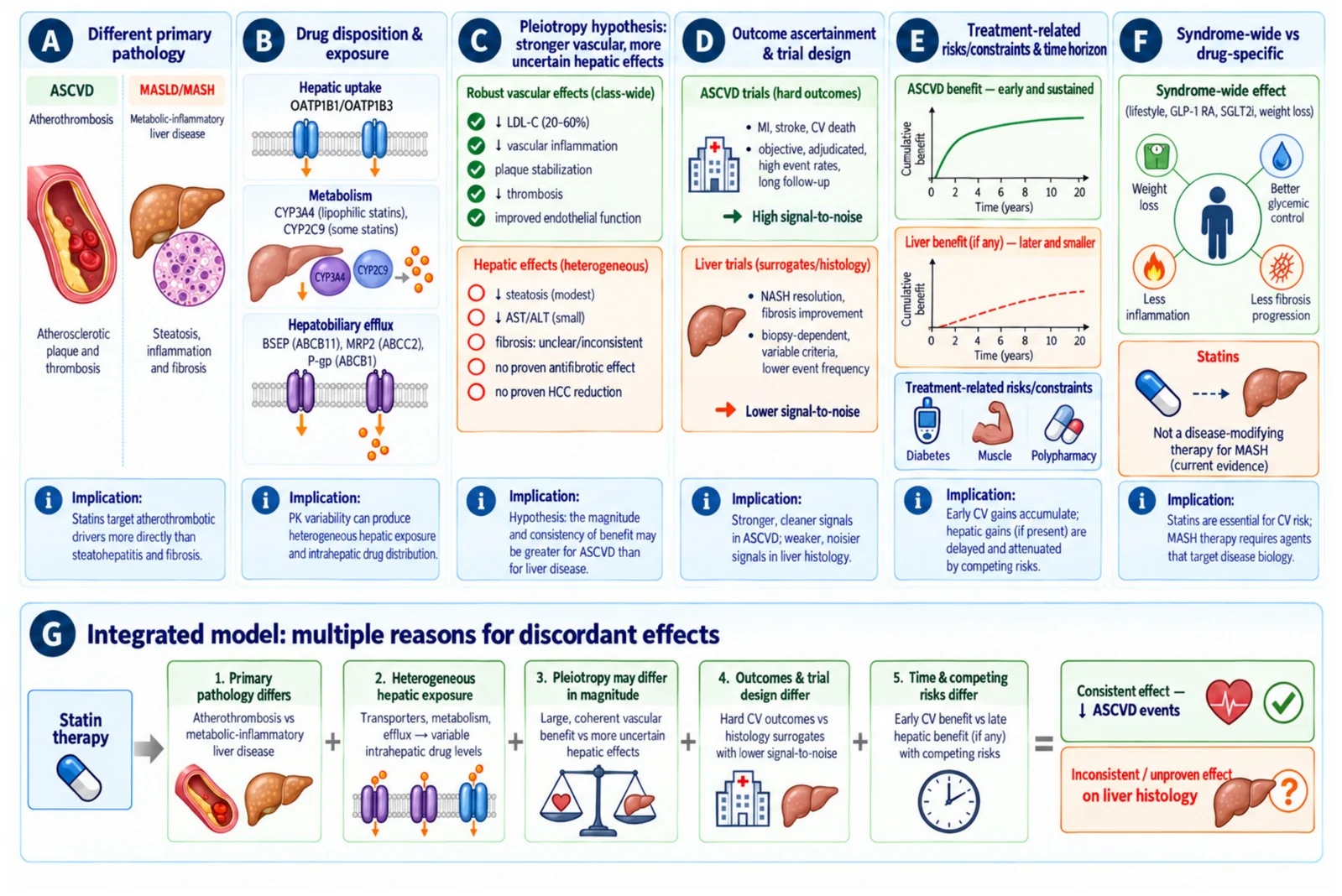
**The sources of discordance: why statins reduce ASCVD events but do not consistently improve liver histology in MASLD.** The apparent discrepancy between the well-established cardiovascular benefits of statins and the uncertain hepatic effects in MASLD may reflect several non-exclusive factors. These include differences in the primary biology of atherosclerotic and steatotic liver disease, variability in hepatic drug disposition and intrahepatic exposure, the hypothesis that statins may exert more pronounced or consistent pleiotropic effects on vascular than on liver-specific pathways, although direct comparative evidence remains limited, differences in outcome ascertainment and trial design, and distinct biological time horizons with competing risks. Statins consistently reduce major atherosclerotic cardiovascular events, whereas their effects on steatosis, fibrosis, liver-related outcomes and hepatocellular carcinoma remain heterogeneous or unproven. Abbreviations: ABCB1, ATP-binding cassette subfamily B member 1; ABCB11, ATP-binding cassette subfamily B member 11; ABCC2, ATP-binding cassette subfamily C member 2; ALT, alanine aminotransferase; ASCVD, atherosclerotic cardiovascular disease; AST, aspartate aminotransferase; BSEP, bile salt export pump; CV, cardiovascular; CYP2C9, cytochrome P450 2C9; CYP3A4, cytochrome P450 3A4; GLP-1 RA, glucagon-like peptide-1 receptor agonist; HCC, hepatocellular carcinoma; LDL-C, low-density lipoprotein cholesterol; MASLD, metabolic dysfunction-associated steatotic liver disease; MASH, metabolic dysfunction-associated steatohepatitis; MI, myocardial infarction; MRP2, multidrug resistance-associated protein 2; NASH, non-alcoholic steatohepatitis; OATP1B1, organic anion-transporting polypeptide 1B1; OATP1B3, organic anion-transporting polypeptide 1B3; P-gp, P-glycoprotein; PK, pharmacokinetics; SGLT2i, sodium-glucose cotransporter-2 inhibitor.

The apparent discordance between observational, randomized and genetic evidence becomes easier to understand once the causal question is clearly defined. An estimand is the specific treatment effect that a study is designed to estimate [71]. In this context, ‘statin use’ is not a single intervention. Prevalent use, treatment initiation, sustained adherence, high cumulative exposure, genetically proxied HMGCR inhibition and systematic statin optimization all represent different treatment contrasts and therefore answer different questions. Active-comparator and new-user designs can improve comparability between treatment groups, but they still estimate effects that differ from those captured by long-term prevalent use [17,18]. Figure 2 summarizes the main sources of this apparent discordance.

This distinction is particularly important because some of the strongest observational associations in MASLD are linked to treatment duration or cumulative statin exposure [13,14,39,40]. Such exposure–response relationships strengthen pharmacological plausibility, but they can also amplify selection bias. To accumulate several years of therapy, a patient must survive, tolerate the drug, remain engaged with healthcare and continue taking it. Cumulative exposure therefore reflects not only the biological effects of the drug, but also a process of patient selection over time.

Observational studies are also vulnerable to several well-recognized sources of bias. Healthy-user effects, immortal-time bias, prevalent-user bias, confounding by indication, survivor selection, healthcare engagement and reverse causation can all produce apparently favourable associations [17,18,19,20,21]. For example, in a study of more than 20,000 new users of lipid-lowering therapy, greater adherence was associated with greater use of unrelated preventive health services [19], illustrating how adherent patients may differ systematically from less adherent patients in ways that are difficult to measure. Immortal-time bias can arise when patients must survive long enough to be classified as exposed [20,21], while falsification-endpoint analyses have shown that unmeasured healthy-adherer behaviour may substantially distort apparent statin effects [22]. Accordingly, very large observational reductions in mortality should increase, rather than reduce, concern about residual bias.

Target-trial emulation can address some of these limitations by explicitly designing an observational analysis to resemble the randomized trial investigators would ideally conduct. This requires alignment of eligibility criteria, treatment strategies, time zero, follow-up, outcomes and causal contrast [21]. Importantly, target-trial emulation is a methodological correction, not a substitute for randomization. Tapper and colleagues emulated the StatLiver trial in Medicare data and obtained essentially neutral estimates for hospitalization, decompensation and death, broadly reproducing the randomized result [57]. By contrast, sequential trial emulation in alcohol-related cirrhosis still suggested a lower risk of HCC with statin use, although residual confounding remained possible [72]. Similar lessons emerge from cancer epidemiology: large apparent statin-cancer associations were substantially attenuated when analyses were aligned with a hypothetical target trial [73,74], consistent with studies showing the influence of selection and immortal-time bias on cancer-survival estimates [75]. However, emulations do not necessarily converge on a single answer, with neutral long-term cancer estimates reported in Hong Kong [76] and a favourable association reported in healthy older adults [77]. Target-trial emulation therefore improves the formulation of the causal question before it improves certainty in the causal answer.

A further issue is the independence of the evidence itself. The same source population may generate multiple publications using different phenotypes, exposure definitions or outcomes. Korean NHIS studies [12,39,40], UK Biobank analyses [36,37] and several Veterans Affairs reports [13,38] should therefore not automatically be interpreted as independent replications. Conversely, a single publication may contain genuinely independent data environments, as illustrated by the UK Biobank, Penn Medicine Biobank and TriNetX components of Vell et al. [36]. Publication count, dataset independence and replication of the same causal estimand are therefore distinct concepts.

Meta-analysis cannot eliminate this underlying causal heterogeneity. Estimates for HCC range from strong apparent protection to statistical neutrality depending on study selection, phenotype definition, exposure classification, and analytical approach [42,43,44]. Low statistical heterogeneity should not be interpreted as low clinical or causal heterogeneity when studies differ in disease stage, exposure window, cancer latency and treatment contrast. Literature may therefore appear statistically consistent while still combining fundamentally different causal questions.

Time horizon is another critical component of interpretation. Cardiovascular benefits of statins may emerge relatively early, whereas fibrosis progression, hepatic decompensation and HCC develop over very different biological timescales [7,11,12,13,32]. A six-month portal-hypertension trial cannot meaningfully test a hypothesis of HCC prevention that may require a decade of exposure and follow-up. Studies may therefore appear discordant simply because they estimate different effects over different periods of disease evolution.

The key question is therefore not which evidence hierarchy should prevail, but whether the design and interpretation of each study are compatible with the causal question being asked. Causal certainty, MASLD-specific relevance, exposure fidelity, independence of the underlying population, and biological latency all need to be considered together when interpreting the statin evidence base.

## 7. Beyond Statin/No Statin: Exposure Fidelity and Pharmacological Precision

A precision approach to statin exposure should currently be viewed as an experimental framework rather than a clinical prescribing algorithm. Its main value is to move beyond the binary classification of ‘statin use’ and consider the sequence from prescription to clinical effect: Prescription → realized exposure → biological/hepatic exposure → target engagement → clinical effect. Each step may be influenced by molecule, dose, adherence, treatment duration, drug transport, co-medication and hepatic reserve.

Observational studies suggest that these dimensions may matter, but they do not yet establish a clinically useful hierarchy. Moderate- or high-intensity statin therapy has been associated with less progression to high-risk FIB-4 [35], higher statin exposure, as estimated using World Health Organization (WHO)-defined daily dosage category, with a lower prevalence of both MASLD and elevated liver stiffness [9], and greater cumulative exposure with lower liver-related risk. By contrast, a Japanese claims analysis found adherence, rather than statin intensity, to be associated with lower incident MASLD [78]. Cumulative exposure is therefore an attractive pharmacological measure, but a difficult causal one, because it simultaneously reflects dose, duration, persistence, tolerance and survival.

Pharmacogenetic and pharmacokinetic data further illustrate why systemic statin exposure cannot be assumed to reflect hepatic exposure. Variants in SLCO1B1, ABCG2 and CYP2C9 influence statin disposition and toxicity [79]. In cirrhosis, an SLCO1B1 loss-of-function variant has been associated with less favourable portal-hypertension phenotypes among statin-treated patients [80], while UK Biobank data link SLCO1B1 variation to LDL response, intolerance and treatment discontinuation [81]. Other studies suggest that SLCO1B1/SLCO1B3 variants may also influence treatment persistence [82].

Hepatic uptake differs substantially between statins. Experimental studies showed variable contributions from OATP1B1, OATP1B3, OATP2B1, membrane permeability and efflux transporters [83]. PET imaging with radiolabelled rosuvastatin further demonstrates that plasma and hepatic concentrations do not necessarily change in parallel [84], and mechanistic modelling can estimate hepatobiliary disposition independently from systemic pharmacokinetics [85]. These findings argue against simple classifications of hepatic efficacy based solely on lipophilicity or hydrophilicity.

Once statins reach their intracellular target, several mechanisms may contribute to their potential hepatoprotective effects. Inhibition of 3-hydroxy-3-methylglutaryl-coenzyme A reductase suppresses the mevalonate pathway, reducing the synthesis of isoprenoid intermediates required for protein prenylation. In particular, reduced geranylgeranylation can modulate the activity of small GTPases, including Rho and Rac, which regulate inflammatory signalling, cytoskeletal organization and cellular contractility [23]. Experimental studies suggest that inhibition of RhoA/Rho-associated kinase (ROCK) and Ras/ERK signalling may attenuate hepatic stellate cell activation, contractility and profibrogenic responses, while also modulating hepatic inflammatory pathways [86]. Statins may also improve hepatic sinusoidal endothelial function through upregulation of Krüppel-like factor 2 (KLF2) and endothelial nitric oxide synthase (eNOS), potentially promoting nitric oxide bioavailability and paracrine mechanisms that favour hepatic stellate cell quiescence [87].

These mechanisms provide a biologically plausible link between statin exposure and potential antifibrotic and portal-haemodynamic effects. However, most of the supporting evidence derives from cell-based experiments and animal models, and findings obtained in human hepatic cells in vitro should not be equated with demonstrated clinical efficacy. Randomized studies in patients with cirrhosis have reported improvements in portal haemodynamics in selected settings [16,64], but these effects do not establish histological fibrosis regression or prevention of major adverse liver outcomes in MASLD. Likewise, favourable associations between statin use and fibrosis-related outcomes in observational cohorts [11,12,36] cannot confirm that these molecular pathways mediate clinically meaningful disease modification. The extent to which such mechanisms are engaged in humans may depend on the statin molecule, achieved intrahepatic exposure, treatment duration and disease stage.

Disease-risk genetics must also be distinguished from treatment-response genetics. Variants such as PNPLA3 are strong prognostic markers of liver disease progression, but evidence that they modify the effect of statin therapy remains exploratory [36,88]. A marker that identifies patients at higher risk does not necessarily identify those more likely to benefit from treatment [89].

Finally, disease stage and co-medication can materially alter achievable statin exposure. Hepatic impairment modifies the pharmacokinetics of rosuvastatin and pitavastatin [90,91], while cirrhosis data confirm that safety depends on dose, molecule and hepatic reserve [56,92]. Resmetirom provides a contemporary example: current US labelling limits concomitant rosuvastatin and simvastatin to 20 mg/day and atorvastatin and pravastatin to 40 mg/day because it increases statin exposure [93]. As liver-directed therapy becomes increasingly layered onto cardiometabolic treatment [94], the statin dose that can actually be delivered may depend on the broader therapeutic regimen.

A true precision-statin strategy will therefore require biomarkers that predict treatment response and tolerability, linking hepatic exposure and target engagement to clinically meaningful outcomes rather than relying only on markers of liver disease severity.

## 8. From Monotherapy to Causal Co-Design: Statins in Layered MASLD Therapy

MASLD treatment is increasingly moving from monotherapy towards layered multidrug strategies. This differs from molecular polyagonism: whereas a single molecule may engage multiple receptors, therapeutic layering combines pharmacologically distinct interventions targeting different components of disease risk. Contemporary care may therefore integrate lipid-lowering therapy, incretin-based or polyagonist treatment, cardiorenal therapy, and liver-directed agents [94,95]. The key question is no longer simply whether these treatments can be co-administered, but what incremental benefit each layer contributes.

Three distinct therapeutic roles can be considered. Efficacy add-on refers to a treatment that further improves an outcome already targeted by background therapy. Risk compensation occurs when one treatment mitigates an adverse effect generated by another. Residual-risk coverage addresses a clinically important domain that remains insufficiently controlled despite otherwise effective treatment. These roles should not be conflated, because each reflects a different causal contribution within a multidrug strategy.

Combination studies illustrate why biological complementarity does not necessarily translate into additive clinical efficacy. In patients already receiving GLP-1 receptor agonists, the addition of efruxifermin produced substantial reductions in liver fat and improvements in several non-invasive markers [96]. By contrast, combinations of semaglutide with cilofexor and/or firsocostat improved selected imaging and biochemical endpoints without clearly establishing additive clinical benefit [97]. Similarly, DUET showed strong activity of TERN-501 but limited incremental benefit from adding TERN-101 across endpoints [98]. More recently, zalfermin and semaglutide were individually active, yet their combination did not provide the expected additional benefit for the primary fibrosis endpoint [99]. These studies reinforce a simple principle: mechanistic complementarity provides a rationale for testing a combination, not evidence that the combination will outperform its individual components.

Risk compensation is equally relevant. Fenofibrate reduced the hypertriglyceridaemia induced by cilofexor/firsocostat [100], demonstrating that an additional treatment may be valuable by offsetting cross-domain toxicity rather than by improving liver disease itself. MIRNA similarly illustrates that favourable hepatic effects can coexist with lipid or apolipoprotein changes in potential cardiovascular relevance [101]. Thus, the need for an additional therapeutic layer may arise from the consequences of treatment in another domain, not only from incomplete hepatic efficacy.

For statins, this framework is particularly useful. Their most defensible role within layered MASLD therapy is currently residual cardiovascular-risk coverage, rather than presumed hepatic additivity. A metabolic therapy may improve obesity, glycaemia, and MASH, and a liver-directed agent may improve MASH or fibrosis, while substantial apoB-mediated cardiovascular risk persists. Observational target-trial emulations of GLP-1RA/SGLT2i therapy in MASLD illustrate that multidrug treatment may confer benefit in some clinical domains without improving others. In patients with MASLD and type 2 diabetes, combination therapy was associated with lower mortality and fewer liver-related outcomes than SGLT2i monotherapy, whereas MACE risk was higher than with GLP-1RA monotherapy [102]. Likewise, a large Danish cohort of patients with type 2 diabetes found that statins were associated with lower atherosclerotic cardiovascular risk than cardioprotective glucose-lowering therapies alone, while combined treatment showed a similar risk to statin monotherapy [103]. These data do not establish pharmacological synergy, but they support the principle that effective treatment in one domain does not eliminate residual risk in another.

Statins are already commonly used as background therapy in contemporary MASH trials. Most participants in the efruxifermin add-on study were receiving statins [96], resmetirom trials reflect similarly layered cardiometabolic treatment [94,95], and pegozafermin analyses have examined safety in patients receiving concomitant statin therapy [104,105]. However, background statin use is rarely randomized or characterized in sufficient detail to quantify its independent contribution. Co-prescription therefore demonstrates feasibility and coexistence, not statin-specific hepatic efficacy.

This distinction is central to the concept of outcome convergence. A multidrug strategy may achieve clinically meaningful benefit across several domains without pharmacological synergy between its components—for example, if one treatment reduces MALO while another reduces MACE. Conversely, improvement in multiple biomarkers does not by itself demonstrate outcome convergence. WAYFIND further illustrates that adding mechanisms does not guarantee greater fibrosis efficacy [106]. Future MASLD trials should therefore characterize background cardiometabolic therapy prospectively, measure actual treatment exposure, and estimate the incremental contribution of each therapeutic layer to clinically meaningful outcomes across domains.

## 9. The Evidence Orphan: When a High-Value Question Has No Owner

The persistent uncertainty surrounding statins in MASLD does not reflect a lack of biological rationale or an absence of clinical investigation. Statins have been studied in NAFLD/MASH, portal hypertension, cirrhosis, and HCC-interception settings [15,16,45,47,48,49,50,51,52,53,54,55,56,63,64,65,66,67,68,69,107,108]. However, these studies have remained fragmented across different disease stages, endpoints, and clinical questions, and have not developed into a coordinated evidence programme capable of determining whether statins modify the major hepatic or oncological outcomes of MASLD. The problem is therefore not that the question has been ignored, but that the available evidence has been generated in disconnected pieces.

We propose that this represents an evidence-orphan problem: an important therapeutic question for which no conventional stakeholder has clear responsibility for generating definitive evidence. The generic status of statins contributes to this problem, but it is only part of a broader structural mismatch involving commercial incentives, specialty boundaries, regulatory pathways, endpoint selection, and the long latency of outcomes such as cirrhosis complications and HCC. Cardiovascular medicine already established the vascular benefits of statins decades ago [7], whereas their potential hepatic and oncological effects emerged later, after the conventional drug-development pathway for statins had effectively ended.

The contrast with resmetirom is illustrative. Resmetirom entered MASH development through a conventional programme with a defined sponsor, prespecified hepatic endpoints, phase 3 histological trials, and a dedicated clinical-outcomes strategy [94,109]. Statins entered the MASLD field from the opposite direction: their cardiovascular indication was already established, while the questions of fibrosis modification, prevention of decompensation, and HCC risk reduction arose only later. These outcomes require prolonged follow-up and cross several specialties, yet there is no obvious sponsor or regulatory pathway responsible for answering the complete question.

Trial registries illustrate the practical consequences of this fragmentation. NCT01987310 planned to enroll 150 participants but was withdrawn before recruitment, with lack of interest cited as the reason [110]. A placebo-controlled pravastatin trial of HCC recurrence enrolled only one participant before termination for low accrual [111]. StatLiver randomized 78 of 162 planned participants, with recruitment difficulties and the COVID-19 pandemic contributing to under-enrolment [52]. The simvastatin HCC-interception trial enrolled 52 of 80 planned participants [69,112], and SACRED recruited 142 participants after an initial programme of approximately 500 [108,113]. These examples should be interpreted cautiously: when the reasons for under-recruitment are not documented, they should not be retrospectively attributed to funding limitations, commercial disinterest, or any other single cause.

Together, these studies illustrate several distinct forms of attrition in evidence generation: failure to initiate, inadequate recruitment, reduction of the planned sample size, unresolved registry status, incomplete reporting, and failure to translate completed experimentation into clinically interpretable evidence. This distinction is important because a registered trial is not equivalent to an initiated trial, an initiated trial is not necessarily completed, and a completed trial is not necessarily reported or clinically informative.

A second problem is the mismatch between clinical relevance and MASLD-specific relevance. Trials conducted directly in MASLD are generally small and rely mainly on surrogate or histological endpoints, whereas studies evaluating hard liver outcomes have largely been performed later in the disease course, in patients with mixed-aetiology cirrhosis [46,51,52,53,54,55,108]. Thus, the absence of definitive randomized evidence in MASLD should not be interpreted as randomized evidence that statins are ineffective.

Importantly, the generic status of statins does not make definitive evaluation impossible. PREVENTABLE demonstrates that very large pragmatic, randomized trials of atorvastatin can be supported through public research infrastructure [114]. PISCATIN provides a liver-specific example by evaluating long-term simvastatin against major clinical outcomes in primary sclerosing cholangitis [115], while MAESTRO-NASH-OUTCOMES shows that event-driven trials using decompensation, transplantation, and mortality endpoints are feasible in contemporary MASH [109]. These programmes do not resolve the statin question in MASLD, but they show that the main barrier is organizational and evidentiary rather than technical.

In this sense, the societal value of answering the statin question is clearer than the conventional ownership of that evidence. The implication is not that MASLD needs another isolated statin trial, but that it requires a coordinated causal evidence programme capable of reconnecting questions that have so far been studied separately.

## 10. Proposed Causal Evidence Programme for MASLD

The statin evidence gap in MASLD is unlikely to be resolved by a single larger randomized trial. At least two different causal questions need to be addressed. One is pharmacological: does a defined statin exposure directly modify MASH activity, fibrosis progression, MALO, or HCC? The other is clinical and implementation-based: does systematic optimization of statin therapy within contemporary MASLD care improve cardiovascular, hepatic, and oncological outcomes compared with usual lipid management? These questions require different study designs and should not be conflated [21,71,116,117,118].

The first question requires a specific molecule and dose under controlled exposure. The second is better framed as the achievement and maintenance of guideline-concordant statin therapy, allowing treatment intensity or molecule to change according to cardiovascular risk, tolerability, drug interactions, and hepatic reserve [2,76,93,119].

This distinction is also important for ethical reasons. Many patients with MASLD already have a clear cardiovascular indication for statin therapy [2,7,9], making long-term placebo allocation inappropriate if the sole purpose is to test a hepatic hypothesis. A direct pharmacological trial is therefore most feasible in carefully selected patients in whom there is genuine clinical uncertainty about whether statin treatment should be initiated, as in STAT-NASH [46]. The methodological starting point should therefore not be “which trial should be conducted?” but instead “which treatment effect needs to be estimated?” [71].

Observational research should follow the same logic. Another comparison of “statin users” versus “non-users” is unlikely to add much unless the hypothetical randomized trial is specified in advance. Eligibility criteria, treatment strategies, treatment assignment, time zero, follow-up, intercurrent events, outcomes, and causal contrasts should all be defined explicitly [21,116,117,118]. In other words, the next observational study should begin with the protocol of the trial it seeks to emulate, rather than with the available database.

In MASLD, several complementary target-trial emulations could address different questions. An initiation analysis could compare statin initiation with non-initiation among statin-naïve patients at a clinically credible treatment threshold. An intensity analysis could compare high- versus moderate-intensity therapy among new users, updating adherence and exposure over time rather than defining future cumulative exposure at baseline. A third design could compare statin initiation with an active non-statin LDL-lowering treatment, such as ezetimibe where clinically appropriate, to explore whether any hepatic effect appears related specifically to HMGCR inhibition rather than to LDL lowering itself [17,18].

These analyses should use a synchronized time zero, new-user designs, time-varying measures of treatment and adherence, competing-risk methods, biologically plausible latency periods, negative-control outcomes, and quantitative assessment of residual bias [17,18,19,20,21,22,57,72,73,74,75,76,77,116,117,118]. Replication should also occur across genuinely independent data sources rather than through repeated analyses of the same healthcare system. The purpose is not to elevate observational evidence to the status of randomization, but to determine whether the reported associations persist when the observational study is designed to approximate the trial investigators would ideally conduct.

The feasibility of this approach has already been demonstrated in chronic liver disease. Target-trial emulation has been used to examine statin initiation and hard liver outcomes in primary biliary cholangitis across independent electronic-health-record environments [58]. These findings cannot be extrapolated directly to MASLD, but the methodological approach is transferable. What is still lacking is an equivalent programme specifically designed around the multidomain outcomes of MASLD.

Observational analyses alone, however, cannot determine whether statins directly modify established MASH biology. A dedicated randomized trial remains necessary. The most informative population would include patients with MASH and F2–F3 fibrosis who are at meaningful risk of progression but do not have a clear cardiovascular indication requiring statin treatment. A predefined statin and dose should be used, with prospective assessment of adherence, achieved exposure, and, where feasible, pharmacokinetic and pharmacogenetic determinants [78,79,80,81,82,83,84,85,88]. Histology and validated non-invasive tests should be assessed longitudinally. Genetics related to liver-disease risk should be analysed separately from genetics affecting statin disposition or response, because a genotype that predicts disease progression does not necessarily predict treatment benefit [79,80,81,88].

The objective of such a trial should remain deliberately focused: to determine whether a defined statin exposure directly modifies MASH or fibrosis. STAT-NASH comes closer to this question than previous statin trials in MASLD, but its planned sample of approximately 70 participants means that it can inform histological efficacy, not MALO, HCC, MACE, or mortality [46].

A second randomized study should address the more immediate clinical question of statin implementation. In patients with MASLD who already have a cardiovascular indication for lipid lowering, the relevant comparison is not statin versus placebo, but systematic statin optimization versus usual-care lipid management. Such an intervention could include proactive identification of untreated eligible patients, protocolized initiation or intensification, assessment of adherence and intolerance, rechallenge or switching when necessary, monitoring of drug interactions, and coordination across hepatology, primary care, diabetology, and cardiovascular medicine.

In this setting, the effect being estimated is not the isolated biological consequence of HMGCR inhibition, but the effect of improving delivery of an already evidence-based cardiovascular therapy within MASLD care. PREVENTABLE demonstrates that pragmatic statin randomization can be embedded within healthcare systems using electronic records, administrative linkage, and remote follow-up [114]. A comparable MASLD study could therefore estimate the benefit of systematic statin optimization without withholding indicated therapy.

Genetic studies should evolve in parallel, but with greater attention to disease stage and outcome. Analyses based only on susceptibility to MASLD are of limited relevance to the treatment question. Future drug-target Mendelian-randomization studies should distinguish disease initiation from progression and examine advanced fibrosis, cirrhosis, MALO, and HCC wherever sufficiently powered phenotypes are available [59,60,61,62,70]. Colocalization, instrument validity, tissue specificity, and the direction of the pharmacological proxy should be reported explicitly. Genetic evidence should be used as supporting causal evidence, rather than as a substitute for pharmacological experimentation.

The choice of clinical endpoints is equally important. MACE, MALO, HCC, broader cancer outcomes, and mortality should remain prespecified, domain-specific outcomes, with both relative and absolute treatment effects evaluated using appropriate competing-risk methods [4,5,6]. A single composite endpoint could be misleading if its most frequent component dominates the result while important differences between cardiovascular, hepatic, and oncological effects remain hidden. Hierarchical or global methods may provide useful secondary summaries, but they should not replace domain-specific estimates [120]. Outcome convergence should therefore be demonstrated by consistent benefit across clinically meaningful outcomes, not created artificially by combining them into a single endpoint.

Operationally, evaluating outcome convergence in clinical research requires several complementary elements: (i) prespecification of at least two clinically meaningful outcome domains, rather than reliance on multiple surrogate biomarkers; (ii) a clearly defined treatment strategy and causal estimand for each domain; (iii) appropriate consideration of baseline risk, disease stage, biological latency, and competing events; (iv) separate estimation of relative and absolute treatment effects, including potential cross-domain harms; and (v) assessment of whether the evidence supporting each effect is sufficiently robust to justify a causal interpretation. In multidrug strategies, the incremental contribution of individual therapeutic components should also be distinguished from the overall effect of the treatment package. Evidence of benefit in one domain should not compensate for uncertainty in another, and apparent convergence should not be inferred solely from a favourable composite endpoint. This approach allows multidomain therapeutic hypotheses to be evaluated without conflating established effects with plausible but unconfirmed benefits.

**Figure 3:**
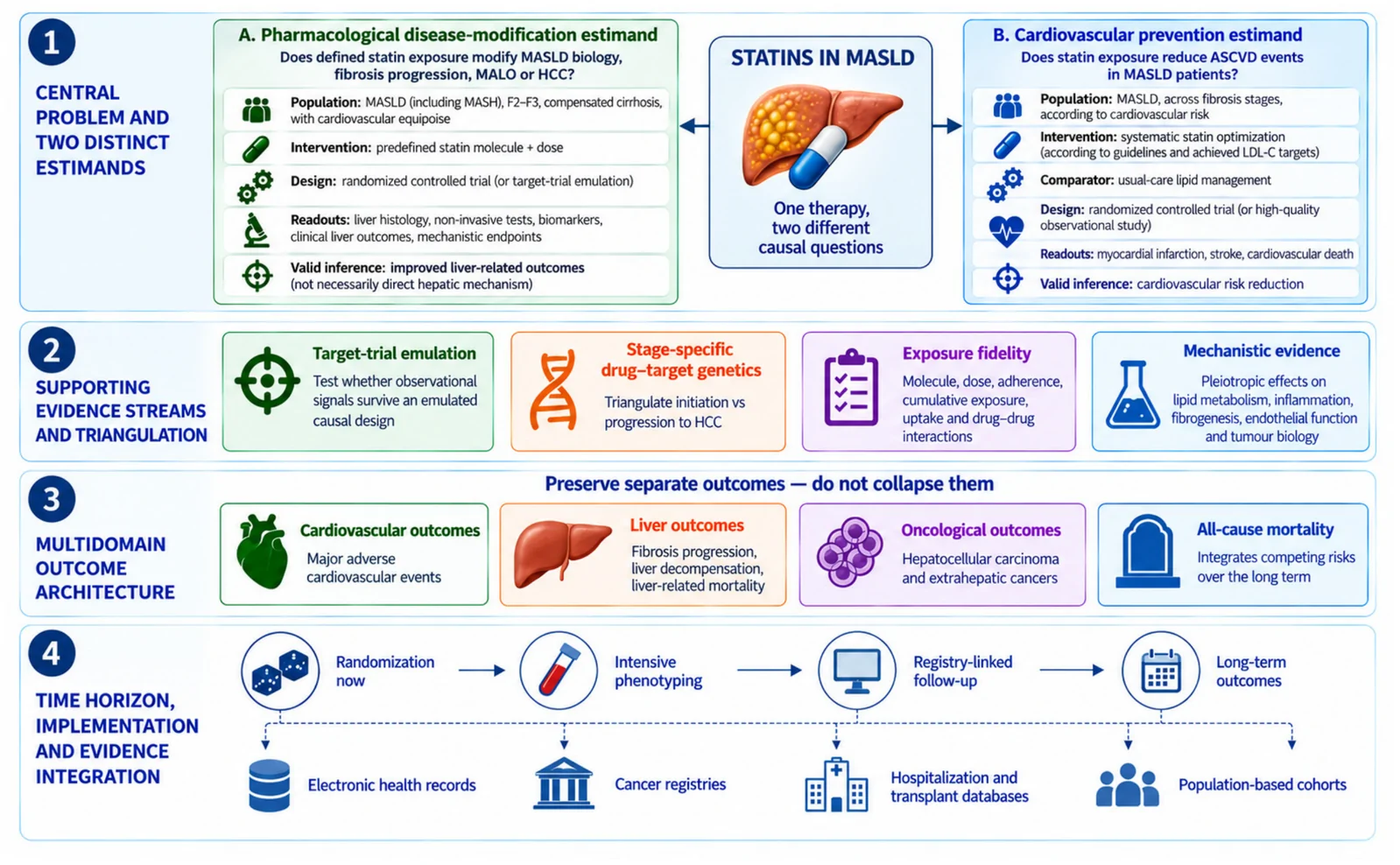
**A causal evidence programme for statins in MASLD.** The statin question in MASLD comprises distinct causal questions that require different study designs. A direct pharmacological approach should determine whether a defined statin exposure modifies MASH activity, fibrosis progression, major adverse liver outcomes (MALO), or hepatocellular carcinoma (HCC), whereas a pragmatic strategy should assess whether systematic optimization of statin therapy improves cardiovascular and other long-term outcomes when treatment is already clinically indicated. These randomized approaches should be complemented by target-trial emulation, stage-specific genetic analyses, exposure-fidelity assessment, and mechanistic studies. Cardiovascular, hepatic, oncological, and mortality outcomes should remain separately evaluated rather than collapsed into a single composite. Because these outcomes emerge over different biological timescales, an efficient strategy would combine initial randomization and intensive phenotyping with long-term registry-linked follow-up using electronic health records, cancer registries, hospitalization and transplantation databases, and population-based cohorts. Abbreviations: ASCVD, atherosclerotic cardiovascular disease; HCC, hepatocellular carcinoma; MALO, major adverse liver outcomes; MASLD, metabolic dysfunction-associated steatotic liver disease; MASH, metabolic dysfunction-associated steatohepatitis.

Long-term follow-up must also reflect the different biological timescales of these outcomes. Cardiovascular events, fibrosis progression, hepatic decompensation, HCC, and extrahepatic cancer do not emerge over the same period. Maintaining an intensive randomized trial for two decades would be impractical, but prolonged follow-up can be achieved through linkage to mortality registries, cancer registries, hospitalization records, transplantation databases, electronic health records, and pharmacy data. Figure 3 illustrates this approach: randomization and intensive phenotyping during the experimental phase, followed by registry-linked follow-up over 10–20 years. Long-term follow-up of randomized statin trials such as ASCOT demonstrates the feasibility of this strategy [121], while contemporary MASH programmes show that adjudicated clinical liver outcomes can be captured reliably [109].

In summary, MASLD does not need a single definitive statin trial, but a coordinated evidence programme addressing complementary questions: rigorous target-trial emulation to test the robustness of observational associations; a focused biological RCT to determine whether statins directly modify MASH or fibrosis; pragmatic randomization of statin optimization where cardiovascular therapy is already indicated; stage-specific genetic studies providing complementary causal evidence; and prolonged follow-up across cardiovascular, hepatic, cancer, and mortality outcomes. The necessary methodological components already exist [46,58,59,60,61,62,70,109,114,116,117,118,120,121]. What remains missing is their integration around the long-term outcomes that matter to the same patient.

## 11. Conclusions

Statins exemplify a central evidence asymmetry in MASLD. Their cardiovascular benefit is firmly established and should be implemented whenever clinically indicated, regardless of whether a direct hepatic effect is ultimately demonstrated. By contrast, observational studies consistently associate statin exposure with slower fibrosis progression, fewer major adverse liver outcomes, lower HCC incidence, and reduced liver-related mortality, but these findings have not yet established causal liver disease modification.

The next step is therefore not to redefine statins as liver-directed therapy, but to test the hepatic hypothesis using study designs aligned with the specific treatment effect of interest: direct randomization where statin initiation remains clinically uncertain, pragmatic optimization where treatment is already indicated, rigorous target-trial emulation, and prolonged follow-up across cardiovascular, hepatic, oncological, and mortality endpoints. On current evidence, statins should be regarded as a candidate, rather than a demonstrated, outcome-convergent therapy.

More broadly, the statin story illustrates a principle that extends beyond a single drug class: in a systemic disease such as MASLD, therapeutic value should ultimately be judged by its impact on the major competing outcomes that shape a patient’s lifetime prognosis, rather than within isolated organ-specific endpoints.

## Figures and Tables

**Table 3:** **Clinical use and unresolved evidence gaps for statins across the MASLD spectrum. **Established cardiovascular indications are intentionally separated from unproven hypotheses of liver disease modification. The table distinguishes current clinical practice from plausible additional benefits and unresolved questions at each stage of MASLD.

MASLD Stage/Clinical Setting	Statin Use in Clinical Practice	Potential Liver Benefit	Key Considerations/Evidence Gap
**F0-F2 MASLD**	Treat according to cardiovascular risk. MASLD itself is not an indication for statin therapy.	Possible reduction in disease progression, but direct hepatic disease modification remains unproven.	Evidence is mainly observational or based on small RCTs. Standard statin prescribing principles apply.
**F3/advanced fibrosis**	Treat when a cardiovascular indication exists.	Observational evidence suggests slower fibrosis progression and fewer liver-related events.	Causal benefit remains unproven; definitive randomized MASLD evidence is lacking.
**Compensated cirrhosis/high HCC-risk MASLD**	Use when cardiovascularly indicated; compensated cirrhosis is not a contraindication to statin therapy. Statins should not be initiated specifically for HCC prevention.	Possible reductions in portal pressure, decompensation, mortality and HCC risk.	Evidence is heterogeneous and largely observational for HCC prevention. Dose, statin type, hepatic reserve and drug interactions are relevant; chemoprevention remains unproven.
**Decompensated cirrhosis**	Cardiovascular benefit must be balanced against a narrower therapeutic window.	Possible benefit in selected patients, but results are inconsistent.	Safety becomes central; high-dose simvastatin should be avoided/extrapolated with caution. Optimal molecule and dose remain uncertain.
**F2-F3 MASLD receiving resmetirom**	Continue cardiovascular lipid-lowering therapy when indicated.	Independent hepatic benefit of statins remains unproven.	Resmetirom increases exposure to several statins and may limit achievable statin doses.

Abbreviations: HCC, hepatocellular carcinoma; MASLD, metabolic dysfunction-associated steatotic liver disease.

## Data Availability

No new data were created or analyzed in this study. Data sharing is not applicable to this article.

## Supplementary Materials

The following supporting information can be downloaded at: https://www.mdpi.com/article/10.3390/ijms27219407/s1. Refs [9,11,12,13,14,33,35,36,37,38,39,40,41,122,123,124,125,126,127,128,129,130,131,132,133,134,135] are cited in SM.

## Abbreviations

apoB, apolipoprotein B; ASCVD, atherosclerotic cardiovascular disease; FIB-4, Fibrosis-4 index; HCC, hepatocellular carcinoma; HMGCR, 3-hydroxy-3-methylglutaryl-coenzyme A reductase; MACE, major adverse cardiovascular events; MALO, major adverse liver outcomes; MASLD, metabolic dysfunction-associated steatotic liver disease; MASH, metabolic dysfunction-associated steatohepatitis; NIT, non-invasive test; SGLT2i, sodium-glucose cotransporter-2 inhibitor; VCTE, vibration-controlled transient elastography.
