# Supplementary material for: Statins and the Lifetime Outcomes of MASLD: From Evidence Asymmetry to Outcome Convergence
Source: Int J Mol Sci. 2026 Oct 22;27(21):9407. doi: 10.3390/ijms27219407 (PMC13763035; doi:10.3390/ijms27219407)
Supplement: Supplementary file 1 [file ijms-27-09407-s001.zip › ijms-4616519-supplementary.pdf]

Statins and the lifetime outcomes of MASLD: from evidence asymmetry to outcome convergence

Javier Crespo, Marta Alonso-Peña, Carolina Jiménez-González, Lorena Cayón-González, Paula Iruzubieta

Supplementary Table S1. Genealogy of the major observational evidence on statins and liver outcomes. This table examines the genealogy of the major observational datasets to distinguish repeated analyses of the same or overlapping source populations from replication across separate data-generating populations. This assessment was performed specifically for this Review and is not intended as a study-quality grading system.

Evidence family / dataset	Representative studies	Population	Main outcome(s)	Relationship between studies / interpretation
**Korean NHIS**	Yun 2025; Kim 2026; Kang 2026 [12,39,40]	Korean national insurance cohorts	Liver-related events, cirrhosis, HCC, mortality	Studies derive from the same national insurance ecosystem; individual overlap cannot be excluded. They should not be regarded as three fully independent replications.
**UK Biobank**	Tran 2020; Vell 2023; Yang 2025 [36,37,122]	UK Biobank	Liver disease, HCC, mortality	Repeated use of the UK Biobank source population. These analyses address different questions but should be considered one main data environment.
**Scottish PCCIU**	Tran 2020 [122]	Scottish primary-care database	Liver cancer	Separate source population from the UK Biobank analysis reported in the same publication.
**Penn Medicine Biobank**	Vell 2023 [36]	US biobank/EHR	Liver disease, HCC, liver-related mortality	Separate data-generating population from the UK Biobank component of the same study.
**TriNetX**	Vell 2023 [36]	Large federated EHR network	Liver disease, HCC, liver-related mortality	Separate data environment from the UK Biobank and Penn Medicine Biobank components.
**VA metabolic liver-disease cohort**	Suzuki 2025; Karachaliou 2026 [13,38]	US Veterans Affairs	Cirrhosis, primary liver cancer	Analyses arise from essentially the same parent VA cohort and evaluate different outcomes; they should not be counted as independent replications.

**VA VOCAL / cirrhosis cohort**	Kaplan 2019; Mahmud 2022; Chapin 2023 [123-125]	US Veterans with cirrhosis	Decompensation, ACLF, mortality	Studies use the same Veterans Health Administration/VOCAL infrastructure, with substantial population overlap likely.
**VA HCV Clinical Case Registry**	Mohanty 2016 [126]	HCV-related compensated cirrhosis	Decompensation, mortality	Uses the same national healthcare system as other VA studies but a different disease-specific registry, period and source cohort.
**Rotterdam Study**	Ayada 2023; Pustjens 2026 [9,33]	Population-based Rotterdam cohort	Steatosis/fibrosis phenotypes, liver stiffness	Recruitment periods and source population overlap substantially; these studies should be interpreted as related analyses from the Rotterdam Study.
**PERSONS biopsy cohort**	Ayada 2023 [33]	Biopsy-proven MASLD	Histological steatosis, MASH and fibrosis	Separate biopsy cohort from the Rotterdam component reported in the same publication.
**VCTE-Prognosis Study Group**	Zhou 2024 [11]	International multicentre MASLD cohort	LSM progression, liver-related events, mortality	Separate multicentre population from the other major observational datasets.
**MUSC primary-care EHR**	Schreiner 2024 [35,127]	US primary-care cohort	Progression to high-risk fibrosis	The two analyses arise from the same centre, period and closely overlapping cohorts; they should be interpreted as one evidence family.
**NHANES 2017–2018 VCTE**	Ciardullo 2021; Polpichai 2024 [128,129]	US NHANES VCTE cycle	Advanced fibrosis / at-risk MASH	Both analyses use the same NHANES 2017-2018 VCTE cycle and therefore should not be considered independent replications.
**NHANES mortality**	Hajifathalian 2022; Ng 2022 [41,130]	US NHANES linked mortality cohorts	Overall and cancer-related mortality	Both studies derive from NHANES and likely contain substantial individual overlap.
**Swedish ESPRESSO**	Sharma 2024 [130]	Swedish histopathology-linked cohort	Severe liver disease, cirrhosis, liver-related mortality	Separate nationwide histopathology-linked population.
**Optum Clinformatics**	Zou 2023 [14]	US claims-based NAFLD cohort	Incident HCC	No overlap identified with the major UK Biobank, VA, Korean NHIS or Rotterdam datasets.
**Advanced biopsy-proven multinational cohort**	Vilar-Gomez 2018 [132]	Multinational F3–F4 MASLD cohort	Decompensation, HCC, vascular events, mortality	Separate multinational biopsy-proven cohort; provides clinically relevant counterevidence to some favourable observational signals.

**Taiwan noncirrhotic MASLD**	Lee 2017 [133]	Clinically noncirrhotic MASLD cohort	Incident HCC	Separate clinical population addressing HCC risk in a predominantly noncirrhotic setting.
**US MASLD HCC case-control cohort**	German 2020 [134]	Small clinical cohort, predominantly cirrhosis	HCC	Separate clinical cohort, although substantially smaller than the population-based datasets.
**Advanced MASH F3/F4 cohort**	Pinyopornpanish 2021 [135]	Advanced MASLD, predominantly cirrhosis	HCC	Separate cohort; the HCC signal predominantly reflects patients with cirrhosis and should be interpreted as stage-specific.

Abbreviations: ACLF, acute-on-chronic liver failure; HCC, hepatocellular carcinoma; LSM, liver stiffness measurement; MASLD, metabolic dysfunction-associated steatotic liver disease; NHANES, National Health and Nutrition Examination Survey; NHIS, National Health Insurance Service; VA, Veterans Affairs; VCTE, vibration-controlled transient elastography.
